# Increase in daily asthma medication sales in association with air pollution levels in Greater Stockholm

**DOI:** 10.1097/EE9.0000000000000256

**Published:** 2023-06-22

**Authors:** Andreas Tornevi, Henrik Olstrup, Bertil Forsberg

**Affiliations:** 1 Section of Sustainable Health, Department of Public Health and Clinical Medicine, Faculty of Medicine, Umeå University, Umeå, Sweden

**Keywords:** Asthma medications, Purchases, Pharmacies, Short-term exposure, PM_10_, Ozone, NO_*x*_, Pollen, DLNM

## Abstract

Daily air pollution levels are known to influence the number of patients with acute asthma. We investigated the short-term effects of air pollution exposure on the daily number of asthma medication purchases in the Greater Stockholm area, Sweden.

**Methods:**

We conducted a time-series study with data on asthma medication purchases and daily mean values of particulate matter ≤10 µm (PM_10_), nitrogen oxides (NO_*x*_), and ozone during 2018–2019. We used nonlinear distributed lag quasi-Poisson regression models to estimate the associations between air pollution levels and medication purchases, adjusting for meteorological variables, pollen levels, day of the week, and long-term trends. The models established linear relationships between air pollutants and the outcome, and potential delayed effects were smoothed with a spline across a lag period of 2 weeks. We applied separate models for each municipality (n = 21) in Greater Stockholm, and calculated pooled estimates to achieve combined results for the whole region.

**Results:**

We observed associations between daily levels of air pollution and purchases of asthma medications, most clearly for PM_10_. The pooled estimates of the relative risks for asthma medication purchases across all 21 municipalities associated with a 10 μg m^−3^ increase in PM_10_ the same day (lag 0) was 1.7% [95% confidence interval (CI): 1.2%, 2.1%], a cumulative increase of 4.6% (95% CI: 3.7%, 5.6%) over one week (lag 0−6), and a 6.5% (95% CI: 5%, 8%) increase over 2 weeks (lag 0−13). The corresponding pooled effect per 10 μg m^−3^ increase in NO_*x*_ and ozone were 2.8% (95% CI: 1.6%, 4.1%) and 0.7% (95% CI: 0%, 1.4%) over 2 weeks (lag 0−13), respectively.

**Conclusions:**

Our study revealed short-term associations between air pollution, especially PM_10_, and purchases of asthma medications.

**What this study adds**

A large part of the population is using asthma medications, but very few studies have analyzed the associations between air pollutants and asthma medication purchases. The observed associations indicate that even moderate levels of air pollution influence a large proportion of asthmatics.

## Introduction

The relationships between outdoor air pollution and asthma exacerbations are well described in the scientific literature, but most studies have analyzed the short-term effects on relatively infrequent and severe outcomes such as emergency room visits and hospitalizations for asthma.^1–3^ The combined estimates from these and similar studies including a meta-analysis generally show around 1%−2% increase in the daily number of cases per 10 µg m^−3^ increase in the concentration of particulate matter (PM_10_ and PM_2.5_), nitrogen dioxide (NO_2_), sulphur dioxide (SO_2_), and ozone (O_3_) on the same day (lag 0), the day before (lag 1), or as their mean value (lag 0−1). The effect of air pollution appears to be stronger in children and elderly.^1–3^ Even in Sweden, where air pollution concentrations are relatively low, short-term increases in the concentrations of PM_10_ have been associated with the daily number of emergency room visits for asthma in Stockholm,^4^ and between unplanned healthcare visits for asthma and PM_10_ in the smaller Swedish town of Visby.^5^

In contrast to hospital admissions and emergency room visits mentioned above, the relationships between air pollutants and less severe asthma exacerbations, which do not require contact with healthcare, are not as abundant in the scientific literature. An increased use of asthma medications can be considered an indicator of less serious asthma exacerbations. However, the short-term associations between air pollutants and the use of asthma medications have been described in a few studies other than symptom diaries. Four Canadian studies have found positive associations between wildfire PM and the daily number of dispensed doses of the asthma medication salbutamol, which is intended to treat respiratory conditions.^6–9^ An increased use of asthma medications in association with elevated concentrations of fine particles has been shown in panel studies involving asthmatics in the United States^10^ and Germany.^11^ Additionally, the daily number of doses of asthma medications administered by nurses in elementary schools in Alaska, United States, was associated with the concentrations of PM_10_.^12^

In this study, we have analyzed the short-term associations between asthma medication purchases and fluctuations in the concentrations of PM_10_, NO_*x*_, and ozone in Greater Stockholm during the period 2018−2019. While emergency visits for asthma are rare and primarily reflect individuals with severe asthma, asthma medications are used by a large group of people to control asthma symptoms. If elevated air pollution levels widely increase symptoms and the use of asthma medications, this should also be reflected in increased sales.

## Methods

### Data collection

We collected data on the daily number of asthma medication purchases among the registered residents in 21 municipalities (with a total population of approximately two million inhabitants) within Greater Stockholm during 2018 and 2019. These municipalities include Botkyrka, Danderyd, Ekerö, Haninge, Huddinge, Järfälla, Lidingö, Nacka, Sigtuna, Sollentuna, Solna, Sundbyberg, Tyresö, Täby, Upplands Bro, Upplands Väsby, Vallentuna, Vaxholm, Värmdö, Österåker, and the City of Stockholm. The included types of medications were as follows: (1) medications for acute asthma symptoms (short-acting beta-agonists; ATC code R03AC); (2) medications for long-term treatment of asthma (long-acting beta-agonists; ATC-codes R03BA, R03DC, R03AK). As a control variable for general patterns of behavior regarding the purchase of prescribed medications, we also included data on medications for high blood pressure (beta-blockers; ATC code: C07).

We obtained data on the concentrations of PM_10_, NO_x_, and ozone (daily mean values) from a centrally located measuring station on the rooftop of a 20-m-high building in Stockholm, representing fluctuations in the urban background (above roof) concentrations. The measurement data from this station were used for all 21 municipalities in the investigated region. Data on the daily pollen concentrations in Stockholm were provided by the Palynological Laboratory at The Swedish Museum of Natural History, describing background concentrations of birch (Betula), grass (Poaceae), alder (Alnus), hazel (Corylus), and mugwort (Artemisia). Daily temperature and relative humidity data in Stockholm were collected from the Swedish Meteorological and Hydrological Institute.

### Statistical analysis

To estimate the short-term associations between the daily asthma medication purchases and the varying concentrations of PM_10_, NO_*x*_, and ozone, we employed quasi-Poisson regression models (multipollutant models). To consider a possible time delay between increasing concentrations of air pollution and purchases of medication, distributed lag nonlinear models (DLNMs) were used. We analyzed purchases of short-acting and long-acting asthma separately, as well as their combined totals. The daily total numbers of purchases made by inhabitants in Stockholm municipality were used to construct the regression model. The model was then applied to each municipality (site) within the Greater Stockholm region to allow for site-specific adjustments for all covariates. Pooled estimates, including estimates from all sites, were calculated to obtain general estimates for the entire region.

The regression models adjusted for seasonal and long-term trends using a penalized spline function with up to 7 degrees of freedom (d.f.) per year. Daily mean values of outdoor temperature and relative humidity were controlled for using penalized splines (maximum 4 d.f.), with both variables representing a two-day rolling mean value describing the weather during the same day and the previous day (lag 0−1). Day of the week effects (including national holidays) on purchase patterns were adjusted for using indicator variables. Additionally, daily purchases of beta-blockers were included in the regression models (penalized spline, max 4 d.f.) to control for general pharmacy purchase activity. Beta blocker purchases were assumed to be uncorrelated with the same-day air quality. Pollen levels (birch, grass, alder, hazel, and mugwort) were adjusted for using an 8-day rolling mean for each type of pollen, fitted with penalized splines (max 4 d.f.) to allow for eventual nonlinear associations.

To estimate the effect of air pollution (PM_10_, NO_*x*_, and ozone) on asthma medication purchases, we applied DLNM functions using 0−13 lags. For this analysis, sporadic missing values in air pollution variables were imputed with linear interpolation. Linear associations between the levels of air pollutions and the medication purchases were estimated, and b-splines with 4 degrees of freedom were used to model the association across the lag-space. The choice of the number of degrees of freedom in the lag-space was determined by comparing a quasi-variant of Akaike information criterion (q-AIC) scores between models using different degrees of freedom (ranging from 4 to 14) for the b-spline.

We performed all analyzes in the programming language R (version 4.1.2, The R Foundation for Statistical Computing, Vienna, Austria) with package mgcv^13^ (for penalizing splines), dlnm package^14^ (for DLNM-functions), and package mv-meta^15^ for calculation of pooled estimates. Regression models were fitted with GAM (generalized additive model) using REML (restricted maximum likelihood). For the meta-analyses, random-effects models were fitted through REML.

## Results

**Figure 1.**
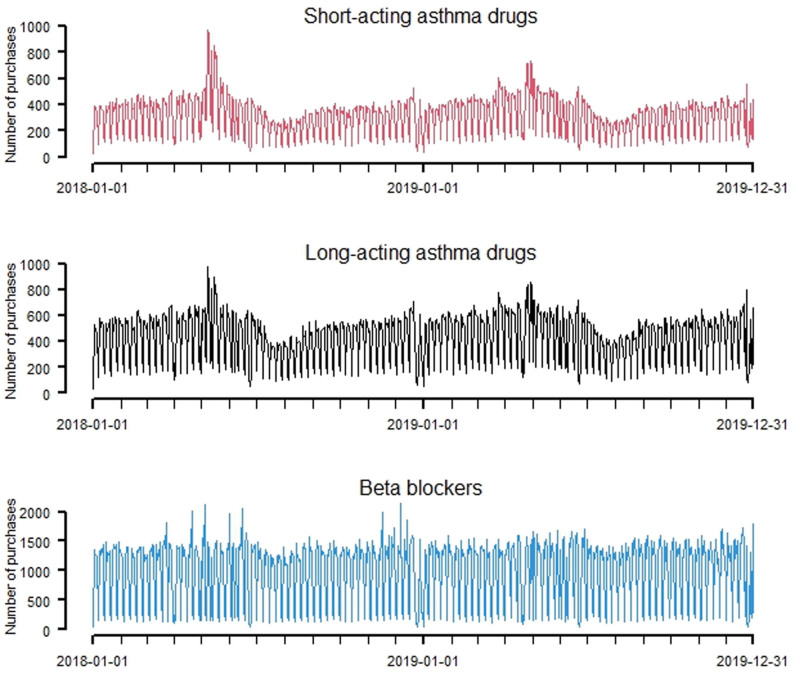
Time series of purchases of medications in Stockholm municipality during the period from 2018 to 2019.

**Figure 2.**
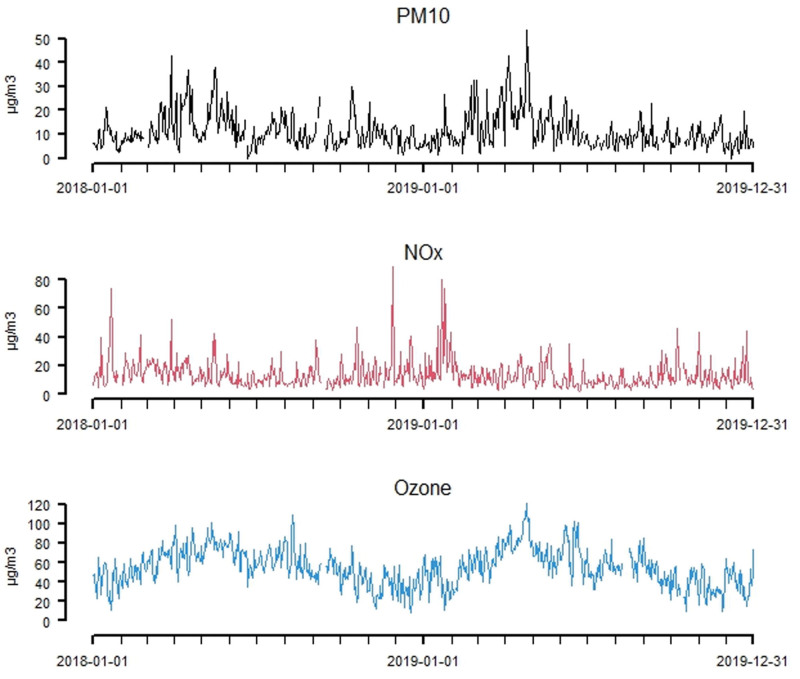
Time series of the daily mean concentrations of PM_10_, NO_*x*_, and ozone in Stockholm during the period from 2018 to 2019.

**Figure A1.**
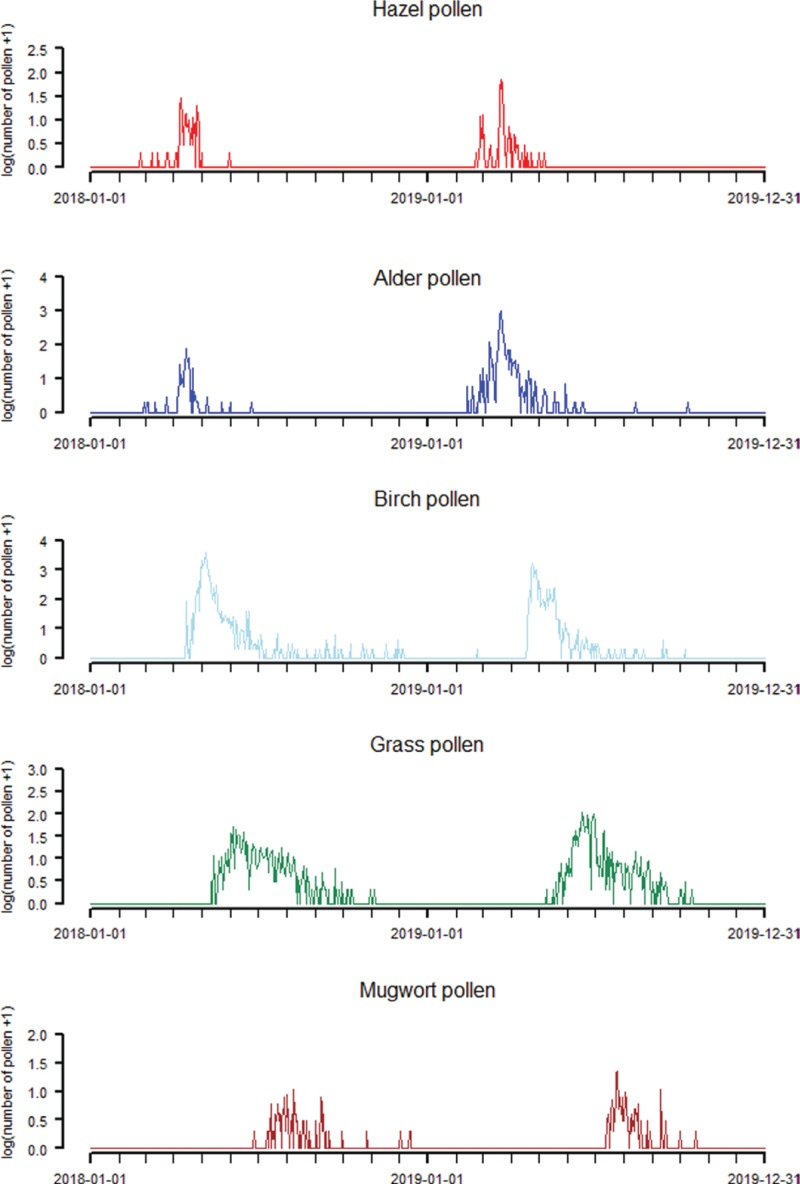
Time series of hazel, alder, birch, grass, and mugwort pollen measured in Stockholm during the period from 2018 to 2019 (log scale).

**Figure A2.**
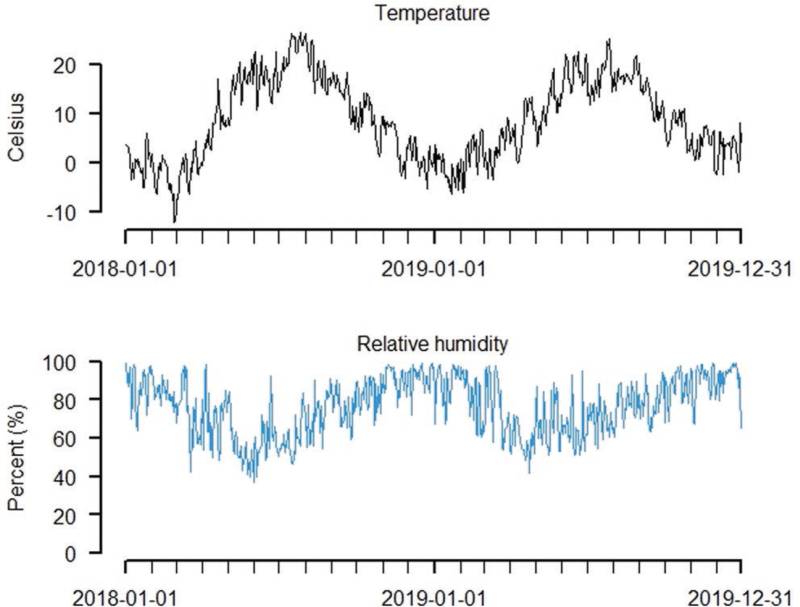
Time series of the daily mean values of temperature and relative humidity measured in Stockholm during the period from 2018 to 2019.

Table 1 presents summary statistics for the concentrations of PM_10_, NO_*x*_, and ozone measured at an urban background site in Stockholm during the 2-year period of 2018−2019. The daily mean values of PM_10_ ranged between 0 and 53 µg m^−3^, NO_*x*_ ranged between 2 and 89 µg m^−3^, and daily ozone concentrations ranged between 8 and 121 µg m^−3^. In Table 2, summary statistics regarding the purchases of the studied asthma medications are presented for each municipality within the Greater Stockholm region. On average, the inhabitants in Stockholm municipality made 763 purchases per day, while the inhabitants in the lowest populated municipality (Vaxholm) made an avarage of 9 purchases per day. Time-series graphs illustrating the purchases of medications (inhabitants in Stockholm municipality) and air pollution variables are presented in Figures 1 and 2. Other covariates used in the models are illustrated in Appendix Figures A1 and A2 (five types of pollen, temperature, and relative humidity).

**Table 1.** Summary statistics (percentiles, mean, and number of missing days) for the 24-hour-mean concentrations of PM_10_, NO_*x*_, and ozone in Stockholm during the period from 2018 to 2019 (µg m^−3^).

	Minimum	25th percentile	Median	Mean	75th percentile	Maximum	Missing
PM_10_	-0.5	6.2	9.2	11.2	14.0	53.3	28
NO_x_	2.1	7.5	10.9	13.6	16.9	89.0	14
Ozone	7.9	42.2	54.9	55.1	66.6	121.40	17

**Table 2.** Summary statistics for the daily purchases of asthma medications (2018−2019).

Municipality	Asthma medication	Minimum	25th percentile	Median	Mean	75th percentile	Maximum
Stockholm	Long-acting	28.0	271.0	507.0	441.2	574.0	975.0
	Short-acting	21.0	196.0	353.5	322.2	415.0	966.0
Botkyrka	Long-acting	3.0	24.0	45.0	41.8	56.0	102.0
	Short-acting	2.0	21.0	40.0	37.4	49.0	121.0
Danderyd	Long-acting	0.0	9.0	16.0	14.9	20.0	38.0
	Short-acting	0.0	5.0	9.0	9.5	13.0	31.0
Ekerö	Long-acting	0.0	7.0	13.0	13.1	18.0	41.0
	Short-acting	0.0	5.0	9.0	9.4	13.0	32.0
Haninge	Long-acting	2.0	25.0	44.0	41.0	54.0	82.0
	Short-acting	1.0	23.0	36.0	34.7	44.0	91.0
Huddinge	Long-acting	2.0	31.3	55.0	50.8	67.0	110.0
	Short-acting	2.0	25.0	42.0	39.9	52.0	127.0
Järfälla	Long-acting	3.0	22.0	40.0	37.2	49.0	90.0
	Short-acting	1.0	17.0	30.0	28.6	38.0	105.0
Lidingö	Long-acting	0.0	13.0	27.5	25.4	35.0	62.0
	Short-acting	0.0	8.0	15.0	15.4	21.0	60.0
Nacka	Long-acting	5.0	27.0	49.0	45.1	60.0	91.0
	Short-acting	2.0	18.0	34.0	31.9	43.0	94.0
Sigtuna	Long-acting	1.0	12.0	21.0	19.8	27.0	55.0
	Short-acting	0.0	10.0	16.0	16.1	21.0	54.0
Sollentuna	Long-acting	3.0	21.0	36.5	34.4	45.0	86.0
	Short-acting	1.0	14.0	24.0	24.2	31.0	92.0
Solna	Long-acting	1.0	22.0	35.0	33.6	44.0	80.0
	Short-acting	2.0	16.0	26.5	26.2	34.0	73.0
Sundbyberg	Long-acting	0.0	13.0	23.0	22.0	29.0	54.0
	Short-acting	1.0	11.0	18.0	17.6	24.0	60.0
Tyresö	Long-acting	0.0	16.0	27.0	25.6	34.0	56.0
	Short-acting	0.0	11.0	19.0	18.6	25.0	57.0
Täby	Long-acting	0.0	23.0	37.0	35.5	47.0	79.0
	Short-acting	0.0	15.0	24.0	24.4	32.0	81.0
Upplands väsby	Long-acting	1.0	13.0	23.0	21.9	29.0	57.0
	Short-acting	0.0	10.0	18.0	17.2	23.0	56.0
Upplands-bro	Long-acting	0.0	7.0	13.0	12.9	18.0	36.0
	Short-acting	0.0	6.0	11.0	10.7	15.0	31.0
Vallentuna	Long-acting	0.0	11.0	18.0	17.6	24.0	44.0
	Short-acting	0.0	8.3	14.0	14.4	19.0	45.0
Vaxholm	Long-acting	0.0	2.0	5.0	5.2	7.0	16.0
	Short-acting	0.0	2.0	4.0	4.3	6.0	14.0
Värmdö	Long-acting	1.0	13.0	23.0	21.6	29.0	52.0
	Short-acting	0.0	10.0	16.0	16.1	22.0	39.0
Österåker	Long-acting	0.0	13.0	23.0	22.2	30.0	53.0
	Short-acting	0.0	10.0	16.0	16.0	22.0	47.0

**Figure 3.**
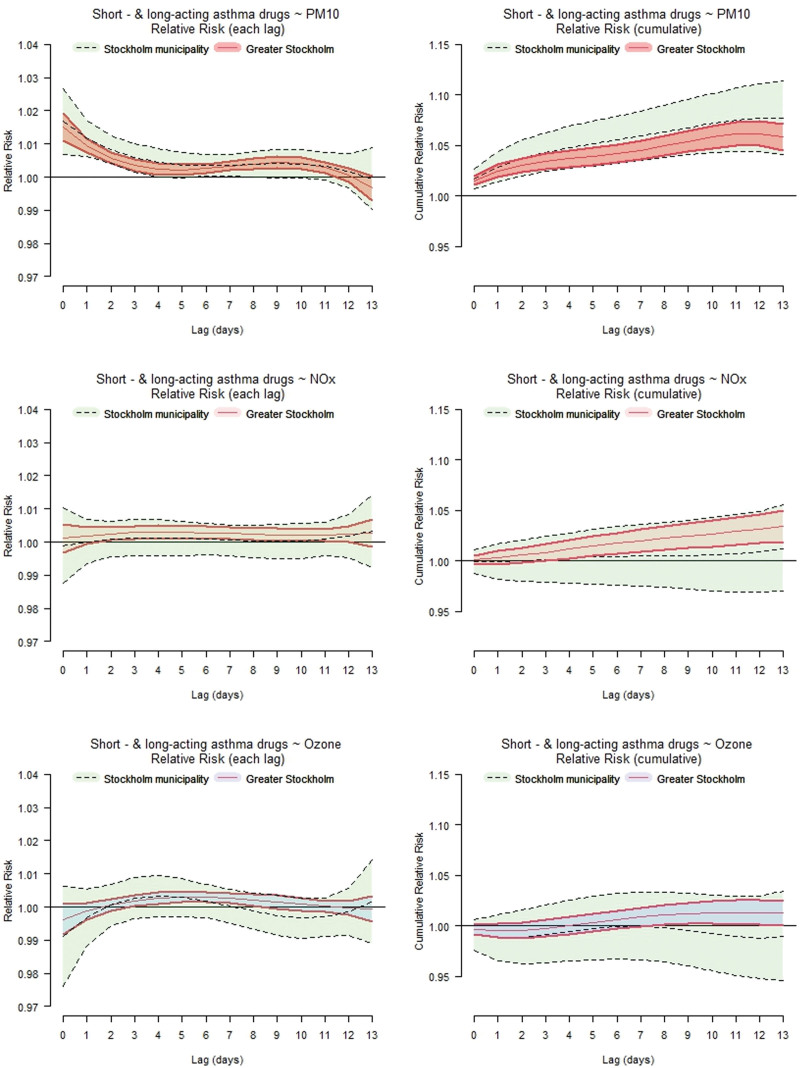
Relative risks (with 95% confidence intervals) on purchases of asthma medications at lag 0−13 associated with a 10 µg m^−3^ increase in the concentrations of PM_10_, NO_*x*_, and ozone for Stockholm municipality (green, dashed lines) and Greater Stockholm (blue/red, solid lines). Left panel illustrates the relative risk in each lag and right panel shows the cumulative effect from lag 0.

There was a clear association between increasing levels of PM_10_ and asthma medications purchased by inhabitants in Stockholm municipality the following 2 weeks, with the strongest effect observed during the first days (Figure 3). Figure 3 illustrates the associations between a 10 µg m^−3^ increase in the 24-hour mean of PM_10_, NO_*x*_, and ozone and the purchases of asthma medications (short- and long-acting combined) as the relative risks at different lags (lag 0, lag 0−6, and lag 0−13) for Stockholm municipality, and for Greater Stockholm (pooled estimate). Figure 3 demonstrates statistically significant effects of PM_10_ on the population in Stockholm municipality and Greater Stockholm, particularly at short lags (lag 0−2). The pooled associations for Greater Stockholm also indicate increased purchases of asthma medication with elevated concentrations of NO_*x*_ and ozone throughout the studied lag period, although the associations are less pronounced.

In Table 3, the health effects in terms of relative risks for asthma medication purchases are tabulated for the same day (lag 0), 1 week (lag 0−6), and 2 weeks (lag 0−13) associated with a 10 µg m^−3^ increase in the levels of PM_10_, NO_*x*_, and ozone. The relative risks are presented for purchases of short-acting, long-acting, and the combined sum of short- and long-acting asthma medications. The relative risks are presented separately for the municipality of Stockholm and the pooled estimates of the 21 municipalities in Greater Stockholm. For the same day (lag 0), purshases of short-acting asthma medication were estimated to increase by 2.2% (95% CI: 1.0%, 3.4%), and purshases of long-acting medication were estimated to increase by 1.6% [95% confidence interval (CI): 0.5%, 2.8%] for a 10 µg m^−3^ increase in PM_10_ levels (Table 3). Similar effects were observed for Greater Stockholm (pooled estimate), and the cumulative effect across the first week (lag 0−6) resulted in an increase of 6.9% (95% CI: 5.7%, 8.1%) in short-acting medications, and 3.1% (95% CI: 2.0%, 4.2%) in long-acting asthma medications.

**Table 3.** Cumulative relative risks for asthma medication purchases associated with a 10 µg m^−3^ increase of PM_10_, NO_*x*_, and ozone in Stockholm municipality, and pooled estimates including 21 municipalities in Greater Stockholm.

	PM_10_ RR [95% CI]	NO_*x*_ RR [95% CI]	Ozone [95% CI]
Asthma medication/Area	Lag 0	Lag 0	Lag 0
Short-acting (Stockholm)	1.022 [1.010, 1.034]	0.997 [0.987, 1.008]	0.988 [0.976, 1.000]
Short-acting (21 municipalities)	1.021 [1.016, 1.027]	1.000 [0.996, 1.004]	0.996 [0.993, 0.999]
Long-acting (Stockholm)	1.016 [1.005, 1.028]	1.000 [0.990, 1.010]	0.997 [0.986, 1.008]
Long-acting (21 municipalities)	1.014 [1.009, 1.019]	1.002 [0.998, 1.006]	1.000 [0.997, 1.003]
Short- and long-acting (Stockholm)	1.019 [1.008, 1.030]	0.999 [0.989, 1.009]	0.994 [0.983, 1.005]
Short- and long-acting (21 municipalities)	1.017 [1.012, 1.021]	1.001 [0.997, 1.004]	0.998 [0.995, 1.000]
	Lag 0-6	Lag 0-6	Lag 0-6
Short-acting (Stockholm)	*1.078 [1.050, 1.108]*	*1.003 [0.977, 1.031]*	*0.992 [0.967, 1.018]*
Short-acting (21 municipalities)	*1.069 [1.057, 1.081]*	*1.016 [1.006, 1.027]*	*0.998 [0.992, 1.004]*
Long-acting (Stockholm)	*1.048 [1.022, 1.075]*	*1.003 [0.978, 1.029]*	*1.003 [0.980, 1.027]*
Long-acting (21 municipalities)	*1.031 [1.020, 1.042]*	*1.017 [1.008, 1.027]*	*1.011 [1.005, 1.016]*
Short- and long-acting (Stockholm)	*1.062 [1.036, 1.088]*	*1.004 [0.979, 1.029]*	*0.999 [0.976, 1.023]*
Short- and long-acting (21 municipalities)	*1.046 [1.037, 1.056]*	*1.014 [1.006, 1.023]*	*1.004 [0.998, 1.009]*
	Lag 0-13	Lag 0-13	Lag 0-13
Short-acting (Stockholm)	*1.106 [1.062, 1.153]*	*1.010 [0.970, 1.051]*	*0.976 [0.942, 1.012]*
Short-acting (21 municipalities)	*1.092 [1.075, 1.110]*	*1.034 [1.017,1.050]*	*1.003 [0.994, 1.012]*
Long-acting (Stockholm)	*1.070 [1.030, 1.112]*	*1.008 [0.972,1.046]*	*1.001 [0.968, 1.034]*
Long-acting (21 municipalities)	*1.049 [1.034, 1.064]*	*1.034 [1.020, 1.049]*	*1.015 [1.008, 1.021]*
Short- and long-acting (Stockholm)	*1.086 [1.046, 1.127]*	*1.010 [0.974, 1.047]*	*0.992 [0.961, 1.025]*
Short- and long-acting (21 municipalities)	*1.065 [1.050, 1.08]*	*1.028 [1.016, 1.041]*	*1.007 [1.000, 1.014]*

Italicized text are listed for all lag 0-6 and lag 0-13 values.

No significant effects of NO_*x*_ and ozone were observed for the inhabitants in Stockholm municipality (Table 3). However, the pooled estimates for NO_*x*_ showed statistically significant combined cumulative increases in purchases of short-acting (1.6%; 95% CI: 0.6%, 2.7%) and long-acting medications (1.7%; 95% CI: 0.8%, 2.7%) the week following a 10 µg m^−3^ increase in the 24-hour mean (cumulative effect lag 0−6) (Table 3). Similarly, there were no significant effects on the number of asthma medication purchases among inhabitants in Stockholm municipality as a result of elevated ozone concentrations, but some of the pooled estimates for Greater Stockholm showed small cumulative effects over the studied lag period, most notably regarding long-acting medications with a cumulative increase of 1.5% (95% CI: 0.8%, 2.1%) for lag 0−13.

**Figure A3.**
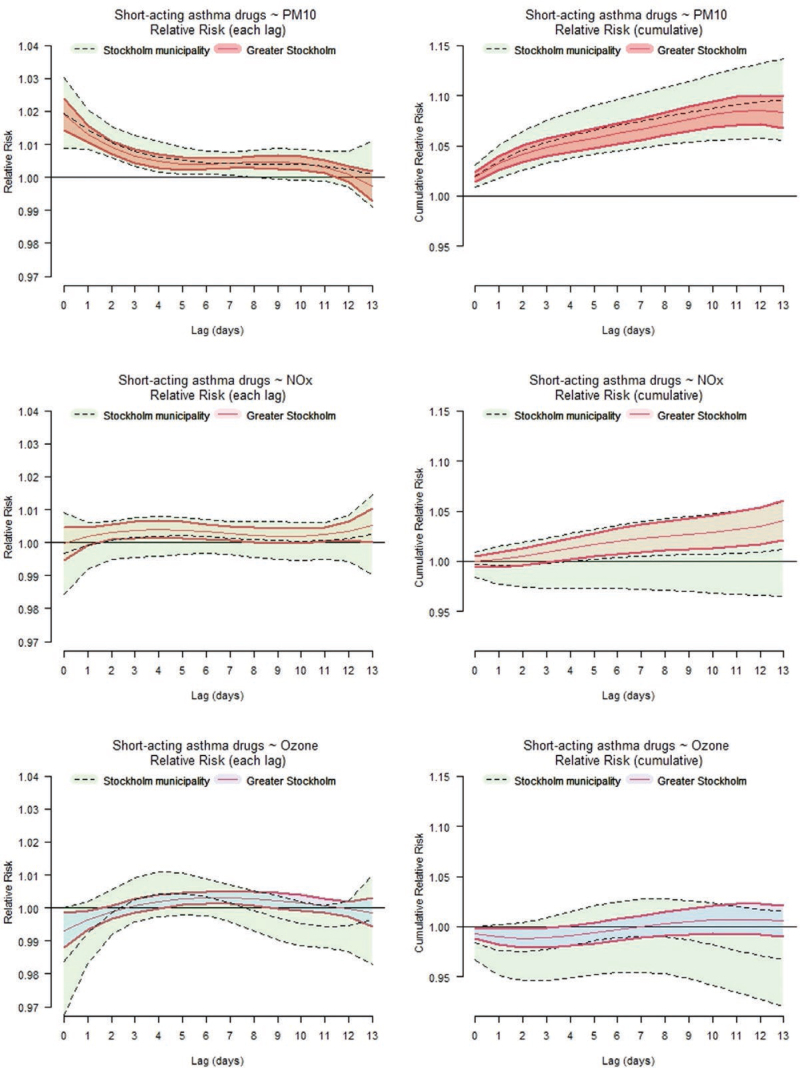
Relative risks (with 95% confidence intervals) on purchases of short-acting asthma medications at lag 0−13 associated with a 10 µg m^−3^ increase in the concentrations of PM_10_, NO_*x*_, and ozone for Stockholm municipality (green, dashed lines) and Greater Stockholm (blue/red, solid lines). Left panel illustrates the relative risk in each lag and right panel shows the cumulative effect from lag 0.

**Figure A4.**
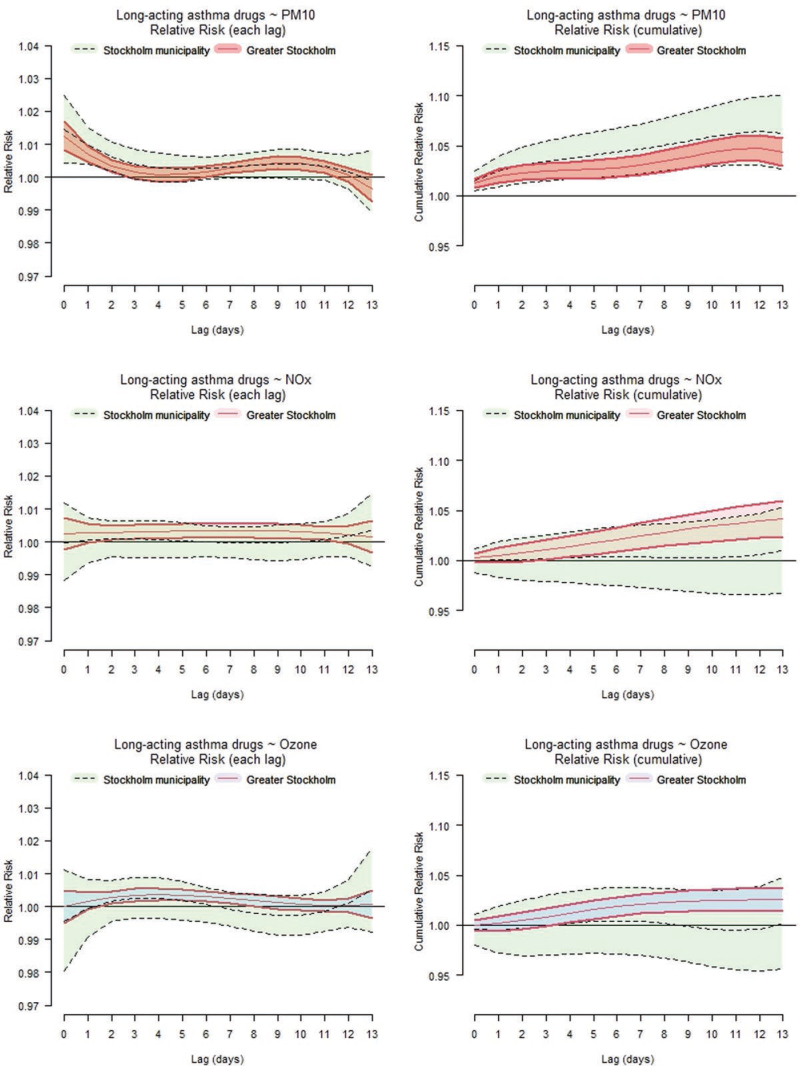
Relative risks (with 95 % confidence intervals) on purchases of long-acting asthma medications at lag 0−13 associated with a 10 µg m^−3^ increase in the concentrations of PM_10_, NO_*x*_, and ozone for Stockholm municipality (green, dashed lines) and Greater Stockholm (blue/red, solid lines). Left panel illustrates the relative risk in each lag and right panel shows the cumulative effect from lag 0.

**Figure A5.**
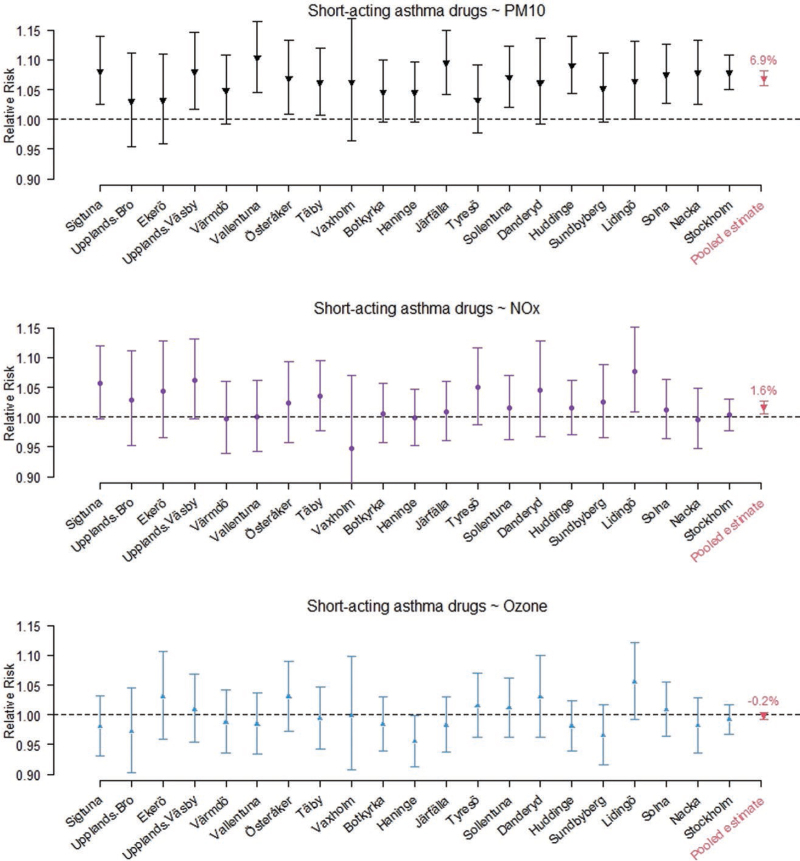
Cumulative relative risks for lag 0−6 on purchases of short-acting asthma medications associated with a 10 µg m^−3^ increase in the concentrations of PM_10_, NO_*x*_, and ozone in 21 municipalities in Greater Stockholm. The pooled estimate for each pollutant, based on the total number of 21 municipalities, is presented to the far right.

**Figure A6.**
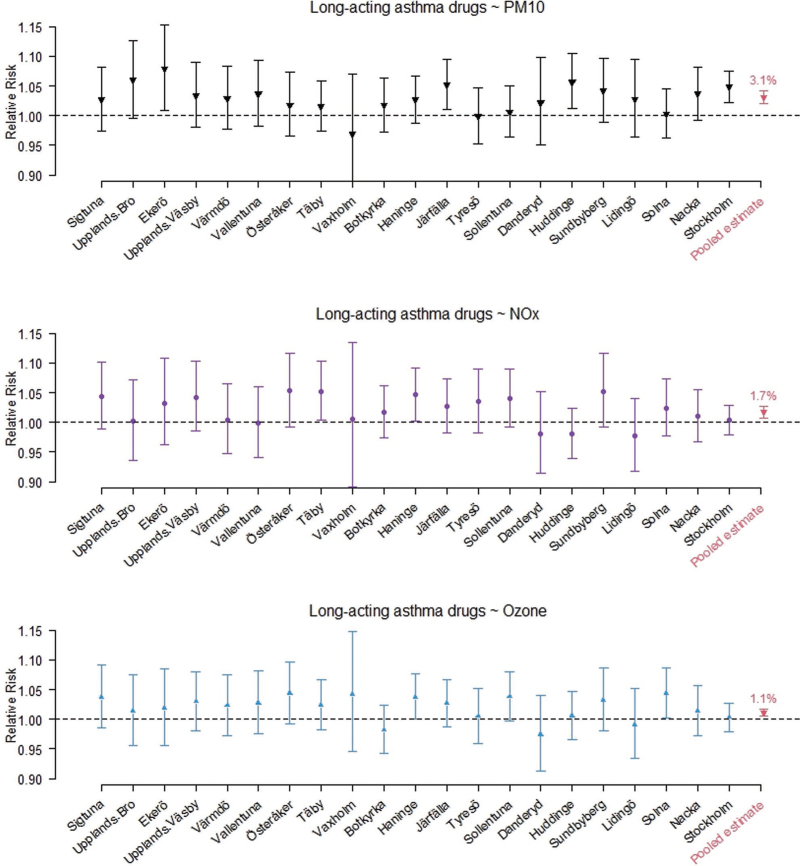
Cumulative relative risks for lag 0−6 on purchases of long-acting asthma medications associated with a 10 µg m^−3^ increase in the concentrations of PM_10_, NO_x_, and ozone in 21 municipalities in Greater Stockholm. The pooled estimate for each pollutant, based on the total number of 21 municipalities, is presented to the far right.

**Figure A7.**
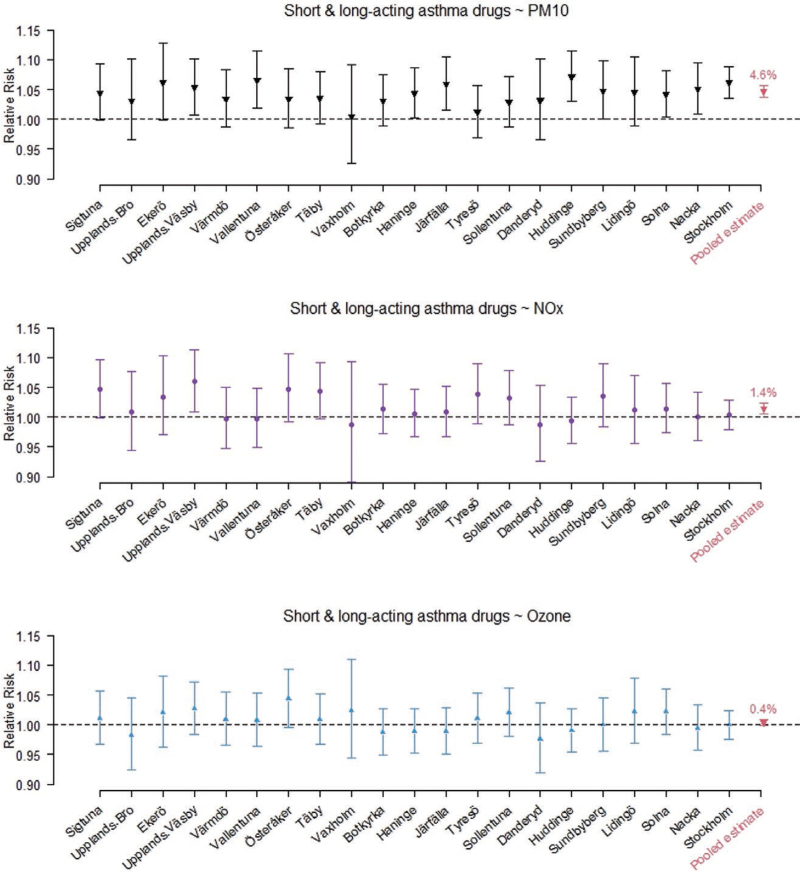
Cumulative relative risks for lag 0−6 on purchases of short-acting and long-acting asthma medications associated with a 10 µg m^−3^ increase in the concentrations of PM_10_, NO_*x*_, and ozone in 21 municipalities in Greater Stockholm. The pooled estimate for each pollutant, based on the total number of 21 municipalities, is presented to the far right.

Figure 3 presents graphs with relative risks for the combined sum of short-acting and long-acting asthma medications associated with a 10 µg m^−3^ increase in the concentrations of PM_10_, NO_x_, and ozone at different lags (separate and cumulative) in Stockholm municipality and Greater Stockholm. In Figures A3 and A4, Appendix A, similar graphs as in Figure 3 are presented, but separating the outcome into purchases of short-acting asthma medications and long-acting asthma medications. The figures reveal almost similar lag structures estimated for short-acting and long-acting medications, indicating a similar purchase pattern in relation to the levels of air pollutants. In Figures A5−A7 in Appendix A, the cumulative relative risks (with 95% CI) associated with a 10 µg m^−3^ daily increase in PM_10_, NO_*x*_, and ozone at lag 0−6 are presented for each municipality, representing both short-acting, long-acting, and the sum of short- and long-acting asthma medications.

## Discussion

### Overall results

The findings of this study reveal that the fluctuations in PM_10_, NO_*x*_, and ozone have varying degrees of impact on the purchases of asthma medications. The most prominent were observed for PM_10_, showing statistically significant pooled relative risks for increased purchases of short-acting asthma medications, which were also significant in the majority of the 21 municipalities (Figure A5), despite the relative short time period used in this study. Additionally, the corresponding pooled estimates of PM_10_, based on the relative risks of increased purchases of long-acting and the sum of short- and long-acting asthma medications in these 21 municipalities, are statistically significant (Figures A6 and A7). It is worth noting that the inclusion of daily sales of beta-blockers as a control variable for general pharmacy activity had minimal impact on the main results.

The pooled estimate of a 7% increase in purchases of short-acting asthma medications associated with a 10 µg m^−3^ increase in the concentrations of PM_10_ on lag 0−6 suggests a greater impact on the medication use compared to what is typically observed for hospitalizations and emergency room visits.^1–3^ Since asthma is common and a quite large proportion of the population is using short-acting asthma medications, this finding suggests that even an increase of rather low particle concentrations has an effect not only on a small number of very susceptible individuals, but on a quite large group of asthmatics. To put into perspective, the relative risks are calculated based on a 10 µg m^−3^ increase in the concentrations of PM_10_, but on the most polluted days, the levels of PM_10_ can reach 30−40 µg m^−3^ above the mean level of 11.2 µg m^−3^ with potentially large effects.

The observed effect on long-acting medications may be unexpected, but it is well known that poor adherence to such medications is a common issue in asthma management. It is plausible that increased symptoms due to air pollution may motivate patients to start also using long-acting medications as well. Additionally, it is likely that patients purchase both types of medications simultaneously.

Contrary to the short-term effects on mortality related to air pollution exposure, with a predominant proportion occurring among the elderly in Stockholm,^16^ purchases of asthma medications are more evenly distributed among the population. Furthermore, the prevalence of asthma declines after the age of 55,^17^ indicating that the elderly population is not driving the observed medication purchase patterns.

### The calculated relative risks

The relative risks calculated for NO_*x*_ and O_3_ in this study were less clear compared to PM_10_. However, elevated concentrations of NO_*x*_ showed pooled statistically significant cumulative relative risks of increased purchases of short-acting, long-acting, and the sum of short- and long-acting asthma medications in the 21 municipalities at both lag 0−6 and lag 0−13, although the relative risks for the largest populated municipality (Stockholm) showed no significant associations. For ozone, there were only statistically significant associations for the pooled estimates regarding long-acting asthma medications at lag 0−6 and lag 0−13, but with a modest relative risk. This is not in line with the results from a review study on emergency department visits and hospitalizations for asthma.^2^ In the region we studied in this paper, high concentrations of PM_10_ from road dust and birch pollen typically occur shortly before the season with the highest ozone levels. As a result, many individuals may have recently purchased their needed medications, which could explain the weaker associations observed for ozone in this study. Consequently, the weaker associations for ozone we found in this paper could therefore be an effect of a storage of asthma medications, where already purchased medications can be used in connection with a worsening related to increasing ozone concentrations. Another possible explanation for the relatively modest associations for ozone could be that the concentrations remained below the threshold value for harmful health effects. However, very few time-series studies have reported a specific threshold value for ozone associated with emergency department visits and hospital admissions due to asthma.^2^

If we compare the relative increase in asthma medication purchases in this study with the corresponding risks estimates in previous studies, the findings are reasonably consistent. In this study, the cumulative relative risks associated with a 10 µg m^−3^ increase in the concentrations of PM_10_ are in the range of approximately 1.03−1.08 for the following week. This is in line with the increased risk of purchasing glucocorticoids and adrenergic inhalants following a 10 µg m^−3^ increase of PM_10_ in seven small- and medium-sized cities in Northern Italy.^18^ Furthermore, when comparing the risk estimates for PM_10_ in this study with the relative risks for salbutamol dispensations associated with a 10 µg m^−3^ increase in PM_2.5_ from forest fires in British Columbia, Canada,^6–9^ the relative risks for PM_10_ calculated in this study were slightly smaller, but of the same order of magnitude. It is worth noting that the risk increase of 25% for medications dispensed for respiratory conditions associated with a 10 μg m^−3^ increase in coal mine fire-related PM_2.5_ in South-eastern Victoria, Australia,^19^ is considerably larger compared to the coefficients for PM_10_ calculated in this study. However, direct comparisons are challenging due to differences in particle composition and exposure conditions. The increased relative risks for asthma medication purchases associated with increases in NO_*x*_ shown in this study, which showed elevated pooled estimates lagged in time and no immediate associations, align reasonably well with a meta-analysis of two ecological studies, which reported a point estimate of 1.008 for respiratory medication sales at lag 8.^20^

### Potential effects related to seasons and pollen

The associations between PM_10_ and asthma medication purchases observed in this study highlight the significant impact of particle exposure on asthma-related problems. However, in the studied region, the sources and chemical composition of PM_10_ are highly related to the seasons. In Stockholm, the content of PM_10_ has a clear seasonal pattern with the highest concentrations measured during springtime, where particles originating from road abrasion contribute up to 90% of the local PM_10_ levels.^21^ In this study, seasonal variations in asthma medication purchases were adjusted using penalized spline functions, which account for general variations in sales primarily driven by pollen exposure but not variations in susceptibility to air pollutants specifically. This study is based on only two years, and the seasonal variations in asthma medications sales associated with air pollution levels have not been analyzed. However, the seasonal variations in health effects in terms of increased mortality associated with short-term exposure to the coarse fraction of PM_10_ (PM_2.5–10_) have been analyzed in Stockholm in two previous studies. Larger effects during springtime in connection with a larger proportion of particles originating from road abrasion were shown in both studies.^22,23^ Statistically significant associations between PM_10_ and asthma medications administered to school children were also shown in Alaska where PM_10_ was largely composed of mineral particles in the coarse fraction originating from road sanding.^12^ It is therefore likely that the seasonal variations in the chemical composition of PM_10_ in Stockholm also affect the asthma medications sales.

In the regression models in this study, the effects of pollen levels (birch, grass, alder, hazel, and mugwort) on asthma medication sales were adjusted for using penalized splines. The incorporation of pollens in the regression models did not significantly alter the estimated effects of the air pollution variables, but it improved model diagnostics, particularly the residual autocorrelation, and enhanced the goodness-of-fit (R^2^). Birch pollen had the most influential impact, which is consistent with findings from a study conducted in France focusing on sales of β2-agonist bronchodilators.^24^ The quantitative and cumulative effects of pollen on purchases of asthma medications in Stockholm, and their possible interactions with air pollutants, will be further explored in future studies.

### Strengths and limitations of this study

A strength of this study is that it includes both PM_10_, NO_*x*_, and ozone, which allows for a fairly comprehensive analysis of air pollutants from different sources. The highest levels of PM_10_ constitutes a marker for road dust, NO_*x*_ constitutes a marker for exhaust, and ozone as a marker for oxidants. Additionally, the study incorporates time lags up to 2 weeks and adjusts for various types of pollen, enhancing the comprehensiveness of the analysis.

However, there are some limitations to consider. First, the regression models are based on a relatively short time period of 2 years, which may limit the generalizability of the findings. A longer study period would provide a more robust assessment of the associations between air pollution and asthma medication purchases. Another limitation is that the air pollutants were measured at a centrally located measuring station in the municipality of Stockholm, which is assumed to represent the surrounding 20 municipalities included in the analysis. This assumption relies on the notion that the temporal variations in pollutant concentrations are relatively consistent throughout the study area. While this may hold true for ozone, which is predominantly influenced by long-distance transport, there may be larger variations in temporal patterns for PM_10_ and NO_*x*_, which are more influenced by local traffic emissions. However, the temporal correlations between air pollutants at different locations in city areas have been analyzed in a few studies. The temporal correlations (monitor to monitor) for O_3_, NO_2_, and PM_10_ within a 100-mile separation distance in seven contiguous states in the United States were calculated with R-values in the range of 0.6−0.8.^25^ Median temporal correlations coefficients in the range of 0.6−0.8 (monitor to monitor) for O_3_, NO_2_, and PM_10_ were also shown based on nationwide data in the United States.^26^

To sum up, the use of one centrally located measuring station is a limitation, but according to the above mentioned studies, the temporal correlations at different sites are reasonable high, and we assume that the traffic intensities show reasonable similar temporal variations at different sites in the study area used in this study. Additionally, the main urban background monitoring station in Stockholm that we have used for this study has previously been used in several time-series studies of air pollution and daily number of deaths or other health outcomes in this region, reporting statistically significant associations.^4,16,22,23^

## Conclusions

The short-term associations between increased concentrations of PM_10_ and asthma medication purchases made by inhabitants in Stockholm municipality, as well as the pooled estimate for Greater Stockholm, are clear and robust, particularly for the purchases of short-acting asthma medications. The associations between NO_*x*_ and asthma medication purchases are less clear, where no effects were shown for inhabitants in Stockholm municipality, but the pooled estimates based on the 21 municipalities within Greater Stockholm were statistically significant for both short-acting, long-acting, and the sum of short- and long-acting asthma medication purchases. Similar for ozone, no statistically significant associations were shown for inhabitants in Stockholm municipality, but the pooled estimates indicated a modest delayed effect, where analyses on purchases of long-acting asthma medications showed higher relative risks than short-acting medications. Overall, there were notable similarities in the distributed lag associations between purchases of short-acting and long-acting asthma medications for different air pollutants. PM_10_ demonstrated a clearer and more immediate effect compared to NO_*x*_ and ozone. The observed associations suggest that even moderate levels of air pollution can impact a significant proportion of individuals with asthma.

## Conflicts of interest statement

The authors declare that they have no conflicts of interest with regard to the content of this report.

## Acknowledgment

We would like to thank Christer Johansson and Sanna Silvergren at the Stockholm Environment and Health Administration for advice on air pollution data, and Agneta Ekebom at the Palynological Laboratory at The Swedish Museum of Natural History for assistance with pollen data.
